# Sol–gel derived B_2_O_3_–CaO borate bioactive glasses with hemostatic, antibacterial and pro-angiogenic activities

**DOI:** 10.1093/rb/rbad105

**Published:** 2023-11-25

**Authors:** Kai Zheng, Faina Bider, Mahshid Monavari, Zhiyan Xu, Christina Janko, Christoph Alexiou, Ana M Beltrán, Aldo R Boccaccini

**Affiliations:** Jiangsu Province Engineering Research Center of Stomatological Translational Medicine, College of Stomatology, Nanjing Medical University, Nanjing 210029, China; Jiangsu Key Laboratory of Oral Diseases, Nanjing Medical University, Nanjing 210029, China; Institute of Biomaterials, University of Erlangen-Nuremberg, 91058 Erlangen, Germany; Institute of Biomaterials, University of Erlangen-Nuremberg, 91058 Erlangen, Germany; Institute of Biomaterials, University of Erlangen-Nuremberg, 91058 Erlangen, Germany; Department of Oto-Rhino-Laryngology, Head and Neck Surgery, Section of Experimental Oncology and Nanomedicine (SEON), Else Kroener-Fresenius-Stiftung Professorship,Universitaetsklinikum Erlangen, 91058 Erlangen, Germany; Department of Oto-Rhino-Laryngology, Head and Neck Surgery, Section of Experimental Oncology and Nanomedicine (SEON), Else Kroener-Fresenius-Stiftung Professorship,Universitaetsklinikum Erlangen, 91058 Erlangen, Germany; Departamento de Ingeniería y Ciencia de los Materiales y del Transporte, Escuela Politécnica Superior, Universidad de Sevilla, 41011 Sevilla, Spain; Institute of Biomaterials, University of Erlangen-Nuremberg, 91058 Erlangen, Germany

**Keywords:** borate glasses, sol–gel processing, glass composition, wound healing

## Abstract

Sol–gel borate bioactive glasses (BGs) are promising ion-releasing biomaterials for wound healing applications. Here, we report the synthesis of a series of binary B_2_O_3_–CaO borate BGs (CaO ranging from 50 to 90 mol%) using a sol–gel-based method. The influence of CaO content in B_2_O_3_–CaO borate BG on morphology, structure and ion release behavior was investigated in detail. Reduced dissolution (ion release) and crystallization could be observed in borate BGs when CaO content increased, while the morphology was not significantly altered by increasing CaO content. Our results evidenced that the ion release behavior of borate BGs could be tailored by tuning the B_2_O_3_/CaO molar ratio. We also evaluated the *in vitro* cytotoxicity, hemostatic, antibacterial and angiogenic activities of borate BGs. Cytocompatibility was validated for all borate BGs. However, borate BGs exhibited composition-dependent hemostatic, antibacterial and angiogenic activities. Generally, higher contents of Ca in borate BGs facilitated hemostatic activity, while higher contents of B_2_O_3_ were beneficial for pro-angiogenic activity. The synthesized sol–gel-derived borate BGs are promising materials for developing advanced wound healing dressings, given their fast ion release behavior and favorable hemostatic, antibacterial and angiogenic activities.

## Graphical Abstract

**Figure:**
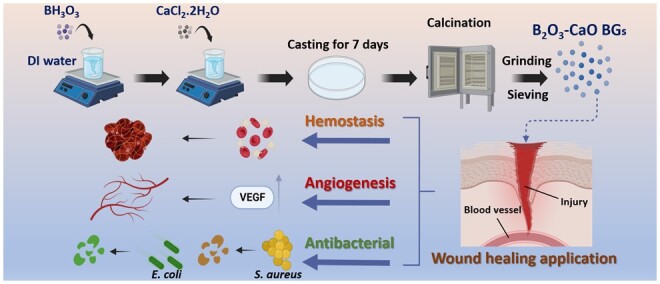

## Introduction

Bioactive glasses (BGs) are synthetic inorganic materials able to bond with bone forming a functional material-bone interface and avoiding encapsulation by fibrous tissues [1]. Since their first development by Prof. Larry Hench in the late 1960s [1], BGs have been clinically used in orthopedics and dental care [2, 3]. BGs are biodegradable and their dissolution products can influence cellular behavior and gene and protein expression, consequently inducing therapeutic effects such as accelerated bone repair [4]. Moreover, the amorphous nature of BGs enables convenient modification of their chemical composition, which allows for the incorporation of biologically active ions [4]. Releasing these ions can bring additional functionalities, such as immunomodulation and antibacterial activity [5]. Owing to these beneficial characteristics, the application of BGs has been extended to soft tissue repair/regeneration, such as wound healing and muscle growth [6].

Silicate-based BGs, such as 45S5 and 13–93 compositions (45SiO_2_–24.5Na_2_O–24.5CaO–6P_2_O_5_ and 53SiO_2_–6Na_2_O–12K_2_O–5MgO–20CaO–4P_2_O_5_ in wt%, respectively), are the most investigated and applied glass compositions in bone-related applications [2, 3]. However, non-silicate, i.e. borate and phosphate BGs, are also of great interest to biomedical applications [7, 8]. In particular, borate BGs, due to their relatively low chemical durability, are expected to dissolve more rapidly than silicate BGs. Rapid dissolution can induce a complete conversion to hydroxyapatite (HA) in acellular simulated physiological solutions [9, 10]. Moreover, borate BGs have shown impressive healing effects on chronic wounds (e.g. diabetic ulcers) [11, 12]. The promising results of the use of borate BGs are likely attributed to their rapid dissolution leading to the favorable and timely release of biologically active ions (e.g. boron ions) beneficial for chronic wound healing [13, 14]. Applications of borate BGs in wound healing are attracting increasing attention [11, 14–16]. However, the investigation of the precise relationship between borate BG composition and wound healing effects remains an important research area.

Sol–gel processing is a convenient approach to produce BGs with controllable (nano)morphology and mesoporosity in comparison to conventional melt quenching [17]. BGs with convenient morphologies, such as nanoparticles [18], nanofibers [19], 3D scaffolds [20], can be produced conveniently using sol–gel approaches, which are thus particularly advantageous for biomedical applications. Moreover, sol–gel-derived BG powders exhibit a larger specific surface area than melt-derived ones due to the intrinsic mechanism of the sol–gel process, consequently leading to more rapid BG dissolution and HA conversion [21]. However, only a few studies have been reported focusing on the sol–gel processing of borate BGs [22, 23]. Borate BGs were produced by the sol–gel method for the first time by Lepry *et al.* [24, 25]. The investigated compositions were intended for bone regeneration and were derived from the classical 45S5 BG composition. These borate BGs exhibited fast dissolution and rapid conversion to bone-like hydroxyl-carbonated apatite. However, these investigated composition systems were complex (four components), challenging the control of degradation and ion release behavior.

The wound healing process covers four overlapping stages: hemostasis, inflammation, proliferation, and tissue remodeling [26]. Most wounds can heal spontaneously. However, disruption of the abovementioned stages may lead to a prolonged wound healing period and consequently result in a chronic wound. On the other hand, application of biomaterials can induce positive effects on one (or more) of the four stages that may accelerate wound healing. BGs have been reported to show hemostatic activity due to released Ca^2+^ triggering the formation of a platelet plug and blood coagulation [27–29]. Calcium also plays a vital role in the late stages of healing, such as facilitating epidermal cell migration to the wound site [28, 30]. BGs with faster dissolution (more rapid release of Ca^2+^) and larger specific surface area (greater contact area with blood) therefore lead to quicker hemostasis and consequently accelerate wound healing [28]. Many events, such as fibroblast proliferation and angiogenesis, are involved in the proliferation stage of wound healing [26]. BGs able to enhance vascularization can thus also greatly accelerate chronic wound healing. Boron (B) has been reported to exhibit enhanced angiogenesis both *in vitro* and *in vivo* [13, 15], thought to be correlated to the impressive wound-healing effects of (melt-derived) borate BGs. However, previous studies usually focused on borate BGs of complex composition systems for wound healing applications [12, 31–34]. Although those investigated borate BGs have shown promising wound healing effects, they are multicomponent glasses, which makes difficult to establish a direct relation between composition and wound healing capability. Developing borate BGs based on simple compositions (two-component glasses) is thus necessary to better understand the composition-wound healing correlation.

In this study, we developed a series of sol–gel derived B_2_O_3_–CaO BGs with CaO mol% = 10, 20, 30, 40 and 50 for wound healing applications. To the best of our knowledge, this work was the first study focusing on the effects of B_2_O_3_–CaO BGs on the wound healing process, including hemostatic, angiogenic and antibacterial activities. We first investigated the influence of CaO content on the morphology, structure, dissolution and ion release behavior of B_2_O_3_–CaO borate BGs and optimized the glass compositions of borate BGs for further investigation. Then the hemostatic and angiogenic activities of the optimized binary borate BGs were evaluated. The correlation between CaO content in B_2_O_3_–CaO borate BGs and their dissolution behavior, hemostatic and angiogenic performances were established. Our results revealed that borate BGs with simple compositions could exhibit controllable dissolution behavior, hemostatic and pro-angiogenic activities favorable for wound healing applications.

## Materials and methods

### Sol–gel processing of borate BGs

**Figure 1.**
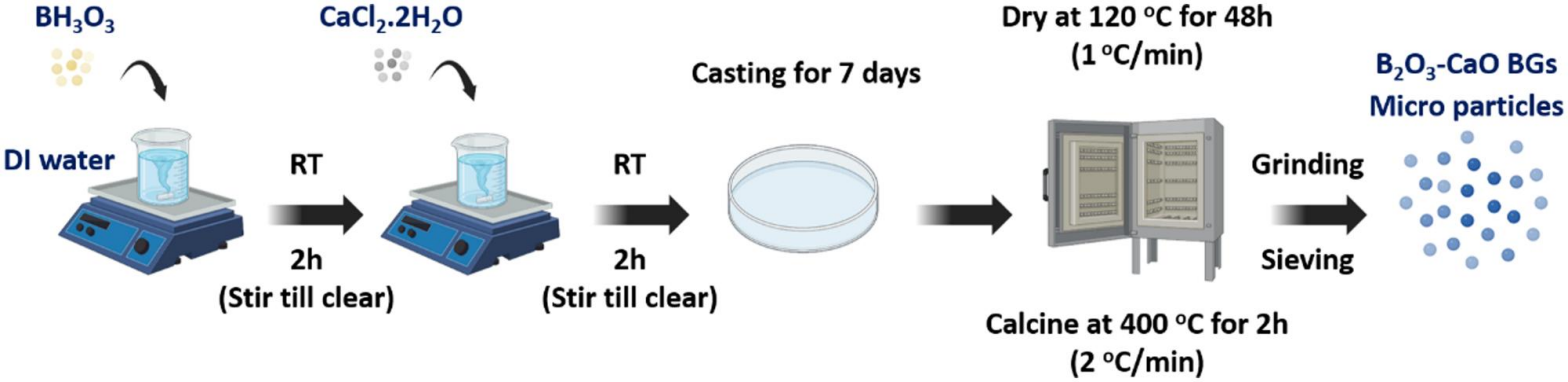
Schematic demonstration of the synthesis procedure of binary B_2_O_3_–CaO BGs.

A series of binary borate BGs with specific glass compositions was synthesized by tuning the CaO/B_2_O_3_ molar ratio, as shown in Table 1. The synthesis procedure is schematically shown in Figure 1. In a typical synthesis procedure of BBG-70 (70B_2_O_3_–30CaO, in mol%), 5 g of boric acid was dissolved in 100 ml of deionized water at room temperature. After 2 h, 2.55 g of CaCl_2_.2H_2_O was added. After stirring for 2 h, the solution was cast in a Petri dish and aged for 7 days at room temperature under a hood. After that, the samples were dried at 120°C for 48 h and calcined at 400°C for 2 h with a heating rate of 2°C/min. The obtained powders were ground and sieved to a particle size below 100 μm. All chemicals were purchased from Sigma-Aldrich without further purification.

**Table 1.** Nominal compositions, codes and precursor information of B_2_O_3_–CaO borate BGs

Codes	Compositions (mol%)	B_2_O_3_	CaO	BH_3_O_3_ (g)	CaCl_2_.2H_2_O (g)
BBG-50	50B-50Ca	50	50	5	5.94
BBG-60	60B-40Ca	60	40	5	3.96
BBG-70	70B-30Ca	70	30	5	2.54
BBG-80	80B-20Ca	80	20	5	1.48
BBG-90	90B-10Ca	90	10	5	0.66

### Physiochemical characterization of borate BGs

The morphology and the elemental distribution of borate BGs were characterized by using a field emission scanning electron microscope (SEM, Auriga, Zeiss, Germany) equipped with an energy dispersive spectrometer (EDS, X-MaxN, Oxford Instruments) system. Transmission electron microscope (TEM, TALOS F200S) was also applied to observe the microstructure of the BGs.

The actual compositions of borate BGs were determined by Inductively coupled plasma atomic emission spectroscopy (ICP-AES, PerkinElmer Optima 5300 DV). The chemical structure of borate BGs was investigated by Attenuated total reflectance-Fourier transform infrared (ATR-FTIR) spectroscopy (IR-Affinity-1S, Shimadzu, Japan) performed at the resolution of 4/cm and by applying 40 scans within the wavenumber range of 400–2000/cm. Glass crystallization was studied by X-ray powder diffraction (XRD, Rigaku, MiniFlex 600, Japan) in the 2θ range of 20°–60° and step size of 0.020°.

### Ion release profiles of borate BGs

ICP-AES analysis was applied to investigate the ion release behavior of borate BGs. Briefly, 100 mg of borate BG powders were immersed in 10 ml of cell culture medium (DMEM, 1 w/v% concentration prepared), placed in an incubating orbital shaker held at 37°C, and agitated at 90 rpm for up to 7 days. The dissolution media were collected at each time point for ion concentration analysis by ICP-AES. The cumulative release of boron and calcium ions was calculated. In order to evaluate the influence of borate BG dissolution products on pH changes, the glass powders were immersed in Dulbecco’s phosphate-buffered saline (DPBS, pH ∼7.4, Thermo Fisher Scientific, USA) at 1 w/v% concentration. After incubation for 24 h at 37°C the pH values of DPBS were recorded.

### Hemostatic activity

Human blood was obtained from healthy volunteers for the plasma coagulation test. The use of human material was approved by the ethics committee at the University Hospital Erlangen (no. 257 14B). Sodium citrate anticoagulated blood was centrifuged at 2500×g for 15 min to obtain platelet-poor plasma (PPP). To investigate the direct coagulation properties of borate BGs, different amounts of BG powders (5, 25, 50 mg) were weighed and placed into tubes before the addition of 100 µl prewarmed H_2_O and 100 µl PPP. The time until coagulation was recorded using MC4 Plus coagulometer (Merlin Medical, Lemgo, Germany). To analyze coagulation caused by the released substances from the particles, the BG powders were placed in 1 ml cell culture medium and incubated overnight with constant shaking at 37°C. Next day, 100 µl of the supernatant was taken and diluted with 100 µl preheated H_2_O. Subsequently, 100 µl PPP was added until coagulation was measured. The coagulation times of pure PPP as negative control (blank) and CaCl_2_ (2.5 mg/ml) as positive control were evaluated.

### Cell culture and preparation

To understand the correlation between glass composition/dissolution behavior and the effects of ions on cells and the wound healing process, *in vitro* tests including WST-8, vascular endothelial growth factor (VEGF) and antibacterial assays, were performed indirectly by preparing extracts of 1 wt%/vol% (10 mg/ml) of each borate glass composition in culture medium. Specifically, the pre-determined amounts of powders were immersed in culture medium for 24 h at 37°C. After 24 h of incubation, samples were centrifuged, and the supernatants were collected for further investigations. Mouse embryonic fibroblast NIH3T3 (CRL-1658, ATCC) cells were cultured in DMEM containing 10% (v/v) FCS and 1% (v/v) of penicillin–streptomycin, l-glutamine and sodium pyruvate at 37°C, 5% CO_2_ and 95% relative humidity.

### *In vitro* cytotoxicity

Cytotoxicity tests of borate BGs were performed using an indirect method. One hundred milligrams of the samples was immersed in DMEM at 1 mg/ml and kept in a shaking incubator at 37°C and 90 rpm. Supernatants (conditioned medium) were taken after 24 h. In parallel 1 × 10^5^ cells were seeded in each well of 12-well plates in triplicates. After 24 h, DMEM was replaced with 1 ml of the separated supernatant for 24 h.

The cell viability was measured by WST-8 assay. The medium was removed from the wells and completely rinsed twice with DPBS. Then, 500 μl of fresh DMEM containing WST-8 (1 vol%) was added to each well. After 4 h incubation at 37°C, the optical density (OD) values were recorded at 450 nm using a microplate reader (Anthos-Phomo, Germany). The cell viability was calculated according to the measured OD values.

### VEGF expression analysis

The angiogenic potential of a material can be evaluated by measuring the secretion of critical biomarkers like the VEGF by cells. Borate BG powders were immersed in cell culture medium at 1% (w/v) and incubated for 24 h. The supernatants were collected and incubated with NIH3T3 cells for 72 h. VEGF secretion was quantified from a 50 μl volume from each experimental group using VEGF mouse enzyme-linked immunosorbent assay kits (RayBiotech, GA, USA) following the manufacturer’s protocol. The OD value of each sample was measured using a multi-mode microplate reader (CLARIOstar^®^plus, BMG Labtech, Germany) at a wavelength of 450 nm.

### Antibacterial assay

The effect of ions released from borate BGs on antibacterial activity was examined via turbidity test using colorimetric measurements. Borate BG powders were immersed in a bacterium medium at 1% (w/v) and incubated for 24 h. The supernatants were collected for the antibacterial test. Additionally, the supernatant of BBG-70 was diluted to different concentrations to evaluate the dose-dependent antibacterial effect of borate BGs. Briefly, the supernatants were cultured with bacteria after 24 h and the OD value of the medium was measured and reported in terms of turbidity. Higher OD values were associated with greater turbidity of bacterial suspension developed from bacterial growth during incubation.

### Statistical analysis

All experiments were performed in triplicate. Data were statistically analyzed using one-way ANOVA. *P < 0.05, **P < 0.01 and ***P < 0.001.

## Results and discussion

### Sol–gel processing of borate BGs

**Figure 2.**
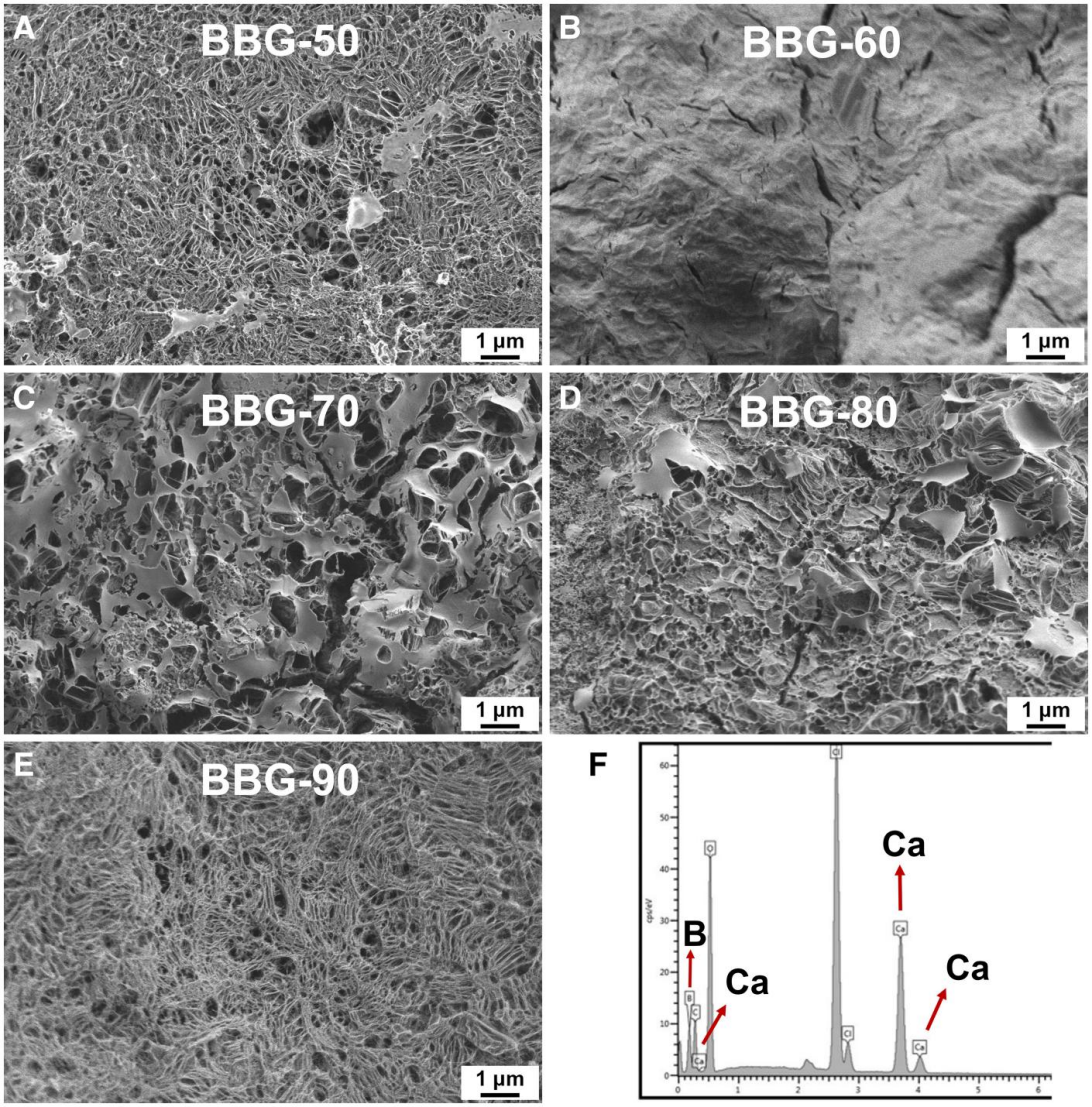
(**A**–**E**) SEM images showing the morphology of binary borate glass powders and (**F**) representative EDS spectrum of BBG-70.

**Figure 3.**
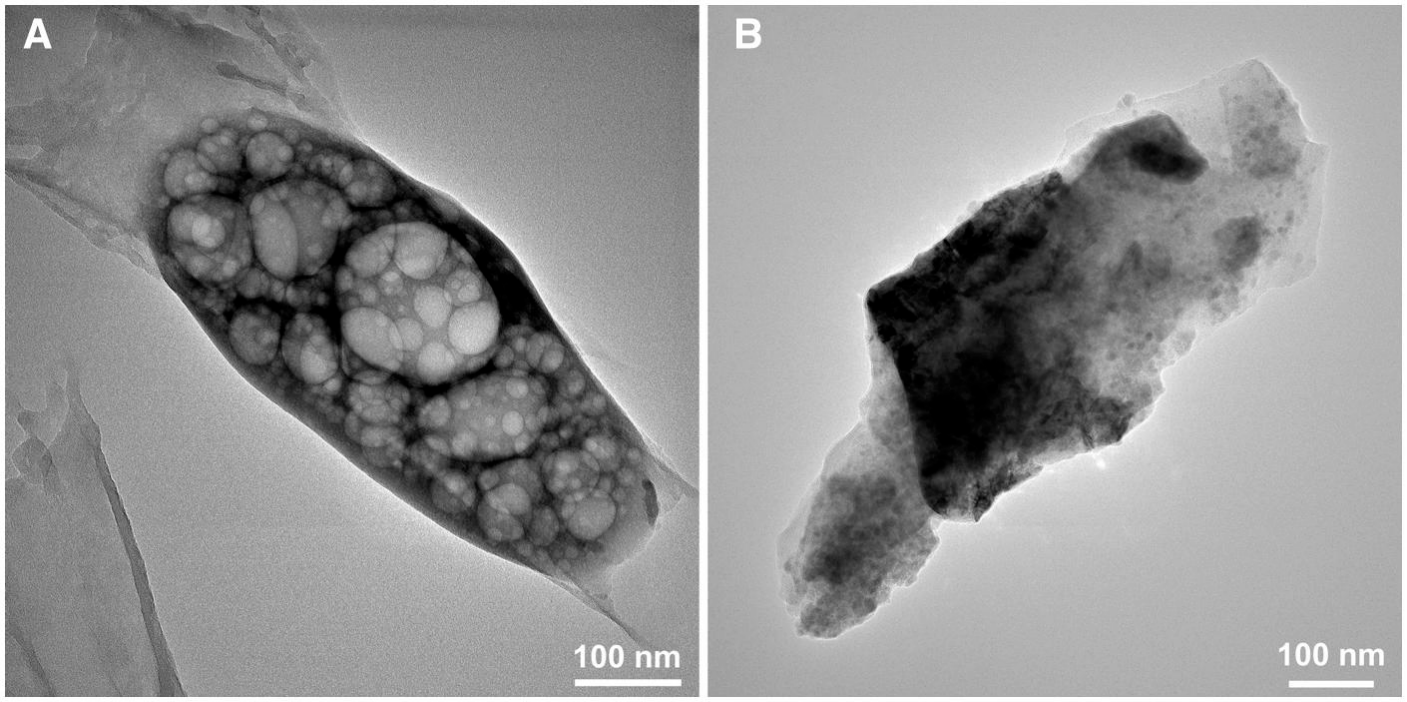
TEM images of representative binary borate BGs: (**A**) BBG-80 and (**B**) BBG-90.

In this study, the calcinated borate BGs were manually ground into small powders with sizes <100 µm for further characterization. Figure 2 shows SEM images of B_2_O_3_–CaO borate BGs and a representative EDS spectrum of BBG-70. As can be seen, all borate BGs exhibited irregular shapes and porous surfaces, although BBG-60 exhibited a relatively dense surface while BBG-90 exhibited a fibrous porous structure. We also observed that these powders exhibited highly hygroscopic properties. The EDS result confirmed the presence of B and Ca in BBG-70. Though EDS could not quantitatively detect B, its presence could be confirmed qualitatively. EDS results of other binary borate BGs also confirmed the presence of B and Ca (data not shown). The presence of Cl was attributed to the use of chloride salt as the Ca precursor. Cl is intrinsically present in the human body, and its minor presence in borate BGs is thus not expected to cause cytotoxicity. Figure 3 shows TEM images of the margin of borate BGs (thin parts). Similar to the observation in SEM images, borate BGs exhibited a micron-sized bulky morphology. The actual chemical compositions of borate BGs were determined by ICP-AES analysis. For the measurement, the powders were digested in acid solution, and the concentration of corresponding ions was subsequently analyzed. The results confirmed the presence of B and Ca in all glasses. The measured molar ratios of B and Ca were consistent with their nominal compositions (Table 1), which was expected because all precursors were aged, dried and calcinated without applying any washing process that could reduce the amount of precursors in the final products.

The main purpose of this study was to investigate the influence of boron content on the biological properties of sol–gel derived B_2_O_3_–CaO BGs. Generally, borate BGs can be converted to HA more rapidly than silicate BGs due to their relatively lower network connectivity. The unique (rapid) dissolution and ion release behavior of borate BGs are important properties for wound healing applications [23]. Increasing interest in the biomedical applications of borate BGs demands the development of novel borate systems with controllable composition and morphology. After the report of the first sol–gel-derived borate BGs in 2015 [24], a series of borate BGs were developed using sol–gel approaches [33, 35, 36]. In this work, we applied a sol–gel process based on a procedure reported in the literature [37]. The main difference in the procedure was the selection of calcium chloride (CaCl_2_) as the source of Ca instead of calcium methoxyethoxide. We did not select calcium methoxyethoxide as the Ca precursor considering its high reactivity and relatively low stability. Although remaining chloride could be detected in the synthesized borate BGs (Figure 2), it is an intrinsic element in the human body and should have minimal negative effects. In addition, the presence of Cl in the composition may reduce the abrasiveness and increase the apatite formation of BGs, which is important for some dental applications, e.g. toothpaste [38].

Most reported borate BGs have been based on borate-substituted ‘45S5’ or ‘13–93’ bioactive glasses [23]. Sol–gel-derived borate glasses of a relatively simple composition system (binary or ternary) have rarely been investigated for biomedical applications. Recently, Lepry and Nazhat developed a series of binary B_2_O_3_–CaO BGs with compositions (100–*x*)B_2_O_3_–(*x*)CaO, *X* = 20, 30, 40, 50, 60 and 70 (mol%). They investigated the effects of B_2_O_3_ content on glass structure, texture and acellular bioactivity [37]. Their results showed that a more porous structure could be observed when the concentrations of B_2_O_3_ were 60 and 50 mol%. Further increase or decrease of B_2_O_3_ led to a relatively denser surface. Our study exhibited a similar trend indicating that the change of B_2_O_3_ content did not lead to a constant change in surface morphology. It is known that the surface microstructure of sol–gel borate BGs depends on their composition and synthesis process [25]. In order to control the morphology of such borate BGs, further studies focusing on the composition and processing parameters are necessary.

### Structure of borate BGs

**Figure 4.**
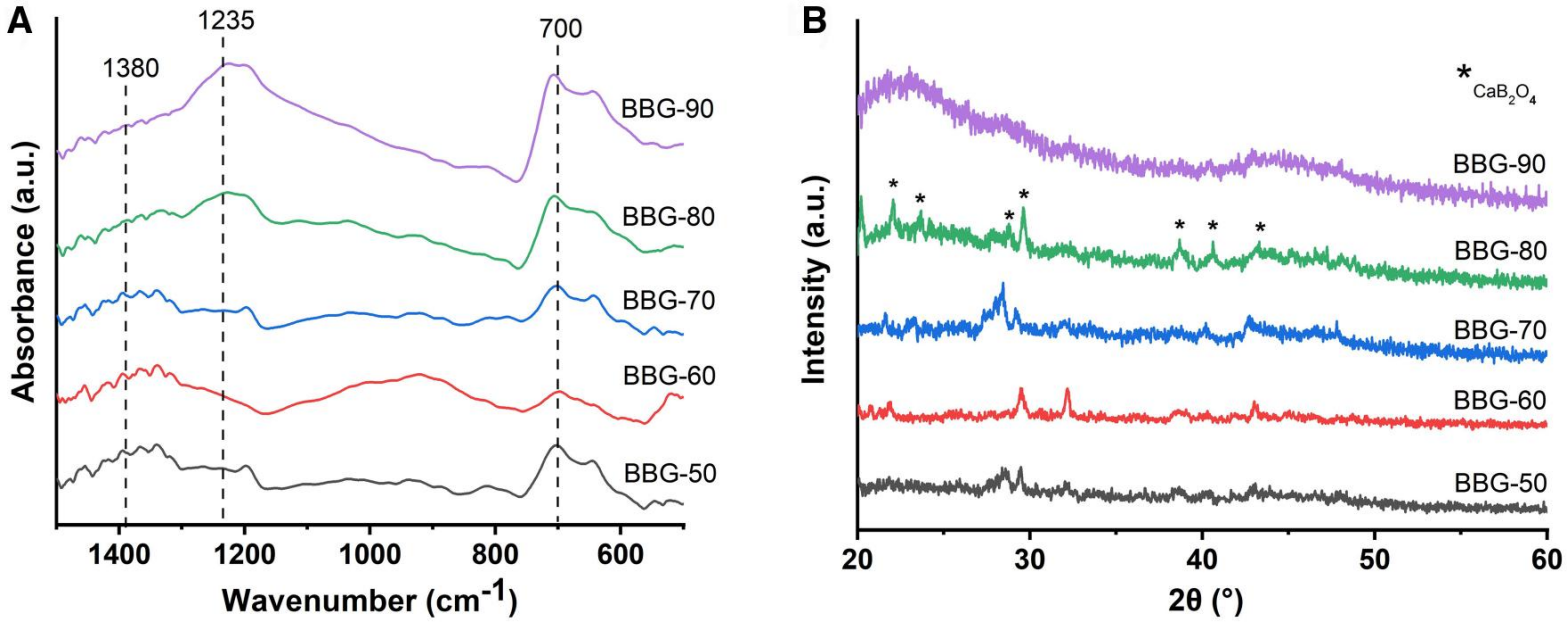
(**A**) ATR-FTIR spectra and (**B**) XRD patterns of the borate glass series synthesized.

The chemical structure of borate BGs was investigated by ATR-FTIR spectroscopy. Figure 4A shows ATR-FTIR spectra of B_2_O_3_–CaO borate BGs. Generally, all borate BGs exhibited three main regions, i.e. the band located between 850 and 1200 cm^-1^ (the B–O stretching of BO_4_ units), the band located between 1200 and 1500 cm^-1^ (the B–O stretching of BO_3_ units), and the band located at ∼700 cm^-1^ (the B–O–B bending of the BO_3_ units) [37, 39]. The presence of these bands confirmed the borate structure of the synthesized BG powders. XRD analysis was performed to understand the influence of CaO addition on possible glass crystallization (Figure 4B). The results showed that only BBG-90 exhibited an amorphous structure, as indicated by the two broad amorphous humps in XRD patterns, while the other binary borate BGs had crystalline phases that could be related to CaB_2_O_4_ crystals [40].

It has been reported that alkaline-earth borate glasses tend to either crystallize at relatively high concentrations of alkaline-earth component or undergo liquid–liquid phase separation at relatively low concentrations [41, 42]. These factors challenge the synthesis of binary alkaline-earth borate glasses, typically B_2_O_3_–CaO glasses. In the mentioned study carried out by Lepry and Nazhat [37], CaO contents up to 73.3 mol% still did not induce crystallization in sol–gel-derived glasses, greatly expanding the composition range of binary B_2_O_3_–CaO BGs compared to conventional melt-quenching compositions [37]. However, our results showed that only BBG-90 (90B_2_O_3_–10CaO, mol%) maintained an amorphous structure (Figure 4B). With the increase of CaO content, crystallization occurred and the formed crystals were probably CaB_2_O_4_. The use of different Ca precursors in the two studies could be the reason causing the reduced composition window of amorphous B_2_O_3_–CaO glasses. Lepry and Nazhat [37] used calcium methoxyethoxide which has been proven to be an appliable calcium source in sol–gel processing since it allows for Ca incorporation into the silica network at relatively low temperatures (i.e. ≤60°C) without affecting the organic phase. Alternatively, we used CaCl_2_ instead of calcium alkoxide as the Ca precursor considering its solubility, stability and relatively low cost. However, the use of CaCl_2_ introduced chlorine in the final product (Figure 2), which may promote crystallization in the resulting borate BGs (Figure 4B). It has been reported that the incorporation of CaCl_2_ can decrease the glass crystallization temperature [43], increasing the possibility of crystallization. In addition, the presence of Cl in the composition may influence the structure of borate glasses. To the best of our knowledge, only a limited number of studies has focused on the influence of Cl on the structure of borate glasses and the exact role of Cl in borate glasses is still not clear. It has been reported that the incorporation of fluorides could change the ratio of 3- and 4-coordinated borate units increasing the 3-fold coordination. In addition, fluorine anions do not enter the coordination surrounding boron atoms but form fluoride and oxifluoride anions with alkaline-earth cations [44]. Cl may also be present as Cl–Ca(n) species in the glass, in an analogous fashion to the related fluoride glasses having F–Ca(n) species [43]. Considering that both F and Cl belong to halogen elements, Cl is expected to play a similar role as F in the structure of borate glasses however this remains to be investigated.

It is known that the molecular fraction of 4-coordinated borate units (N_4_) is related to the content of glass modifiers in binary borate glasses. Although we did not quantitatively analyze the structural changes with variationss of composition, previous studies have analyzed the relation between CaO content and the molecular fraction of N_4_ [37, 41]. It should be noted that the presence of crystalline phases in borate BGs can also affect the morphology and other compositional characteristics. Therefore, it is likely that the morphological and compositional characteristics of the developed borate glasses with various compositions were mainly related to the different molecular fractions of 3- or 4-coordinated borate units and the presence of a crystalline phase, given that the same sol–gel and calcination processing parameters were applied. However, the main objective of this study was to develop the sol–gel synthesis of binary borate BGs and to evaluate their properties related to wound healing applications. The composition-structure-physiochemical property relationship of the borate BGs was not particularly investigated, which should be the target of future studies.

### Composition-dependent ion release behavior of borate BGs

**Figure 5.**
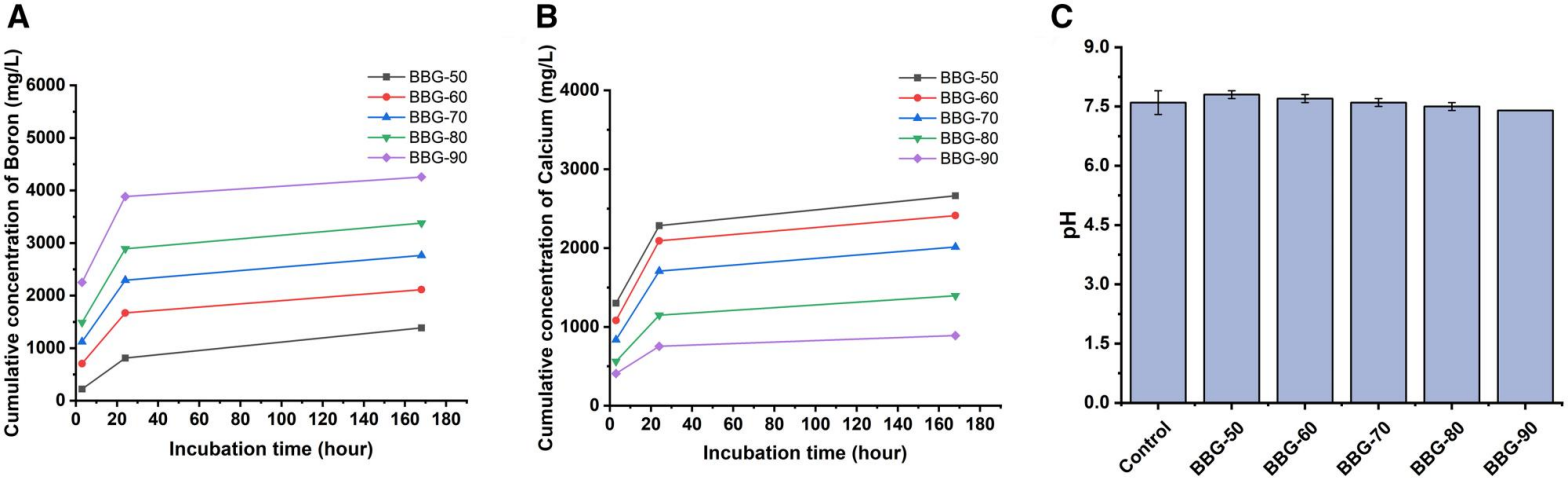
ICP-AES results of binary borate BGs: cumulative concentrations of (**A**) B and (**B**) Ca ions released in DMEM. (**C**) pH values of DPBS containing borate BGs for 24 h at 37°C.

Figure 5A and B show the ion release results of binary borate BGs in DMEM. As can be seen, all glasses exhibited burst release of B and Ca ions within 24 h and sustained release for up to 7 days. Burst release of ions is commonly observed in BGs when they are exposed to biological fluids [45]. When BGs are in contact with aqueous-based solutions, an ion exchange at the material/solution interface takes place and ions are consequently released. However, the surface of BGs can form a Ca-P layer overtime in aqueous-based solutions, which prevents the rapid ion exchange between glass surface and solution, leading to a relatively moderate ion release. In this study, Ca-P formation on the synthesized binary BGs was not considered as we mainly focused on the potential application of borate BGs in wound healing rather than hard (bone) tissue regeneration. Nevertheless, a previous study [37] has shown the rapid formation of Ca-P species on sol–gel derived B_2_O_3_–CaO BGs after exposure to simulated body fluid within 6 h. Therefore, the formation of Ca-P species could be the reason for the moderate ion release after 24 h in DMEM in this study. As expected, borate BGs with a lower content of CaO released lower concentrations of Ca ions while releasing more B ions as they contained higher concentrations of B_2_O_3_. Moreover, the released amount of Ca ions was notably higher than in silicate glasses of similar CaO concentrations. For example, the released amount of Ca ions from 70SiO_2_–30CaO (mol%) BGs (particle size 38–90 μm) at 1.5 mg/ml in simulated body fluid for 168 h has been reported to be ∼100 mg/ml, and equal to ∼666 mg/ml at particle/medium concentration of 10 mg/ml [46]. In our study, the released amount of Ca from BBG-70 (70B_2_O_3_–30CaO, mol%) at 10 mg/ml in DMEM was ∼1800 mg/ml, which was much higher than the released amount of Ca ions from silicate BGs of the same CaO concentration and similar particle size.

Figure 5C shows the pH values of DPBS containing borate BGs for 24 h at 37°C. The pH of all groups (including the control) remained stable at ∼7.5 close to the original pH (∼7.4) of DPBS. The release of Ca ions increases the pH while the release of B ions led to the formation of mildly acidic H_3_BO_3_ or H_2_${\text{BO}}_{3}^{-}$. Because the pH remained stable at ∼7.5, close to physiological pH, the pH variation was not expected to cause significant adverse effects in cell culture studies.

The controlled dissolution of borate BGs is necessary for designing demanded borate BG products for wound healing treatments. It has been reported that the dissolution rate of alkali or alkaline-earth binary borate glasses, unlike binary silicate or phosphate glass systems, usually does not change linearly with increasing modifier content [47, 48]. Velez *et al.* [49] investigated the dissolution kinetics of binary lithium borate glasses in water and buffer solution (pH = 7). The dissolution rates were not linear and the lowest dissolution rate was observed when the molar ratio of Li_2_O was 25–30%. The precise control of the dissolution of borate glasses is challenging, however, it is possible by carefully tuning the glass composition. O’Connell *et al.* [50] achieved a linear release of Sr^2+^ in melt-derived borate glasses by controlling the added content of lanthanum (La) that acted as a modifier. Such control is desired for the applications of BGs in wound healing since it can minimize fluctuation in local concentration that may cause over- or under-dosing of therapeutic ions [51]. Biologically active ions can only exert their therapeutic effects in a specific concentration window [4] and an overdose of ion concentration can even cause toxicity toward cells or tissues. Our results showed that the release of B and Ca ions from binary B_2_O_3_–CaO BGs could be conveniently controlled by regulating the composition, considering the highly composition-dependent ion release behavior.

### Hemostatic activity

**Figure 6.**
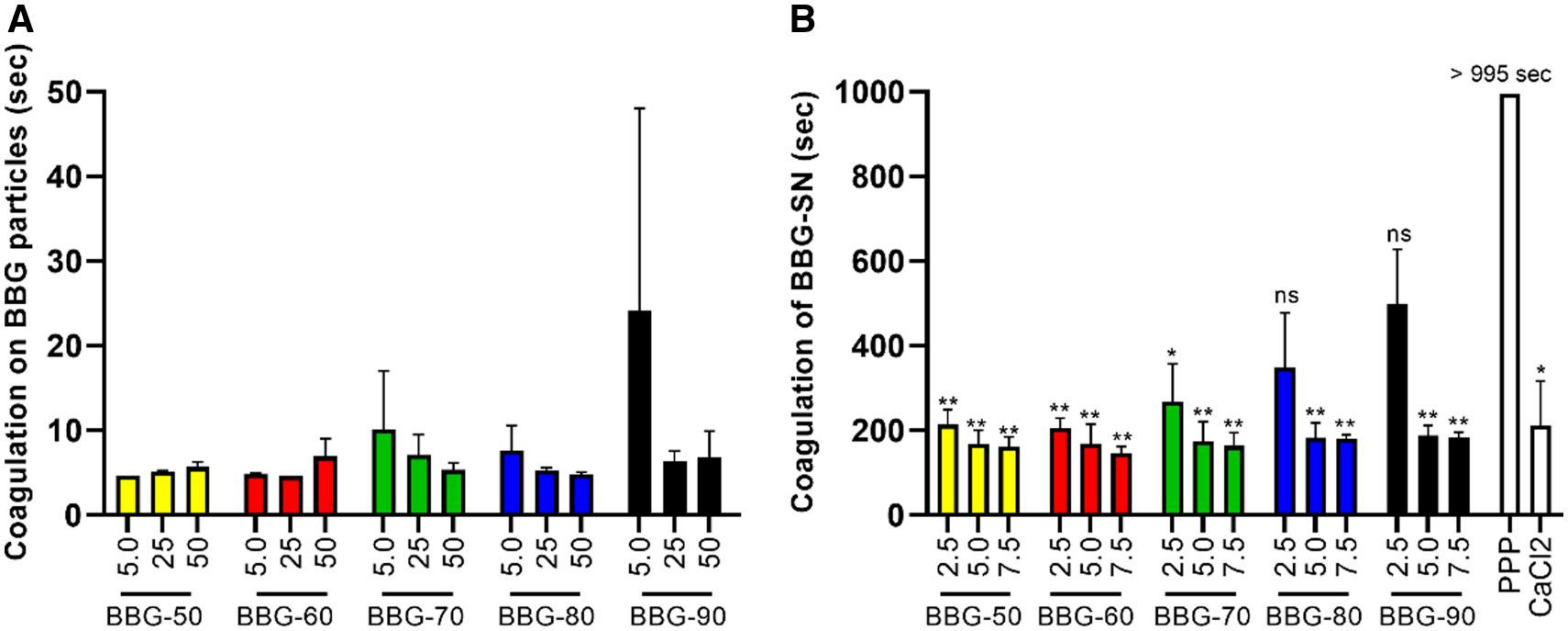
Plasma coagulation time in the presence of (**A**) borate BG powders or (**B**) their supernatants. The mean values of 2 (A) or 3–4 (B) independent measurements with plasma from healthy volunteers with standard deviations are shown.

Figure 6A shows plasma coagulation time in direct contact with borate BG powders. Quick coagulation (a few seconds) was observed in all borate BG groups (compared to the pure PPP control), though the plasma was not firmly coagulated (still wobbly). This could be explained by the covered surface of borate BGs by coagulated plasma preventing further reaction. For 5.0 mg per well, the BG powders containing high borate amounts showed a delayed coagulation time. However, the coagulation time was still faster than for the pure PPP group. As can be seen, BBG-50 and BBG-60 induced faster clotting than BBG-70, BBG-80 and BBG-90, which was probably due to the more rapid dissolution and Ca release of these BBGs (Figure 5). The supernatants of various borate BGs at different concentrations also promoted coagulation (Figure 6B) compared to the pure PPP control, even though the coagulation was slower than the one determined using BG powders directly. Notably, the coagulation time of most borate BGs supernatants was comparable to the coagulation induced by CaCl_2_ (2.5 mg/ml).

The hemostatic activity of BGs has been well recognized [52, 53]. Released Ca ions represent a key factor contributing to the hemostatic performance of BGs as Ca ions (considered clotting factor IV) can function as a cofactor with other clotting agents involved in the activation of both intrinsic and extrinsic pathways of the coagulation cascade [52]. Ca ions can initiate platelet activation and aggregation as well as contribute to the conversion of fibrinogen into stable fibrin clot, leading to enhanced coagulation. The synthesized borate BGs could induce coagulation within 10 s, faster than most microsized silicate BGs reported in the literature [52]. In addition, the induced coagulation by borate BGs was close to the coagulation induced by mesoporous BGs (MBGs) [52]. For example, Nagrath *et al*. [54] reported that Tantalum-containing silicate MBGs could induce fast coagulation (within 20 s) due to the large specific surface area, negative surface charge and released ions. Although the synthesized borate BGs did not exhibit a large specific surface area similar to MBGs, the fast release of Ca ions facilitated coagulation. This was confirmed when we analyzed the supernatants of the BGs for their effect on plasma coagulation. With the supernatants, we also saw that the clotting time was dependent on the dose and type of powder. With increasing amount of borate, the clotting time increased when using supernatants of only 2.5 mg particles (Figure 6B). Similar results have been observed in previous studies. Liu *et al*. [53] investigated the hemostatic activity of silicate 13–93 BG, borosilicate BGs with varying B_2_O_3_ ratios, and borate 13–93B BG as well as the underlying mechanism. They found that coagulation effects decrease with the increase of boron content, which could be explained by the presence of borate ions limiting the action of extrinsic coagulation factors and reducing the interface’s electronegativity of BGs. However, the underlying mechanism by which borate BGs affect coagulation is still not fully understood. Further studies using other techniques (e.g. thromboelastograph) are required in order to elucidate the effects of borate BGs on the coagulation process.

Our results indicated that the binary borate BGs could induce fast coagulation, showing the potential in hemostatic applications. In addition, the hemostatic activity of borate BGs is composition dependent, likely due to the ion release behavior of the glasses. Nevertheless, detailed investigation of the hemostatic properties of borate BGs should be performed to develop advanced wound healing dressing with favorable hemostatic activity.

### *In vitro* antibacterial activity

**Figure 7.**
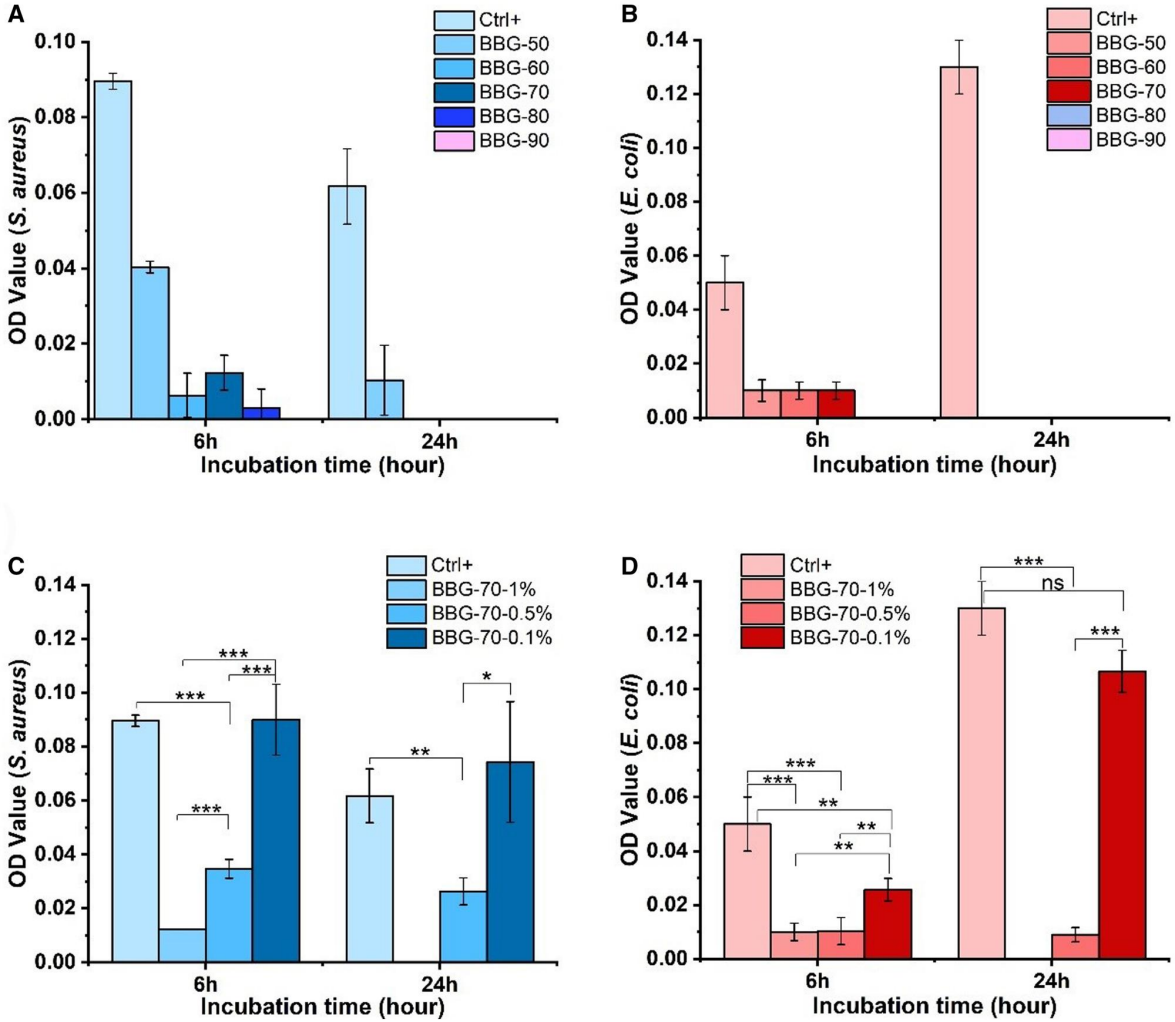
Antibacterial results of binary borate glasses (BBG-50 to BBG-90) examined using turbidity test using colorimetric measurements: (**A**) for 1% (w/v) binary BBG samples against *S.aureus*, (**B**) for 1% (w/v) BBG samples against *E.coli*, (**C**) for BBG-70 1% (w/v), 0.5% (w/v) and 0.1% (w/v) samples against *S.aureus* and (**D**) for BBG-70 1% (w/v), 0.5% (w/v) and 0.1% (w/v) samples against *E.coli* after 6 and 24 h, respectively.

Figure 7 shows the antibacterial results of borate BGs. The OD values of all borate BG groups were reduced in both types of gram-positive and gram-negative bacteria (*Staphylococcus aureus* and *Escherichia coli*, respectively) compared to control values at the first 6 h. The results demonstrated that bacterial activity was inhibited when B ion release increased. The results also revealed that the antibacterial activity of the samples comparatively increased to nearly killing the bacteria in both species when the interaction time of extracts with bacterial suspension rose from 6 to 24 h. However, the most effective inhibition was observed on gram-negative bacteria (*E. coli*). The decreased OD values of samples compared to the control confirmed the growth restriction of both bacterial species by releasing B ions.

Several types of BGs have been shown to exhibit antibacterial activity, which is known to be composition depend. BGs can induce bactericidal activity either by increasing local pH or releasing antibacterial ions (e.g. Ag, Cu) [55]. In our study, borate BGs exhibited effective antibacterial performance, perhaps induced by the released ions. BBG-90, BBG-80, BBG-70 and BBG-60 showed significantly stronger antibacterial activity than BBG-50, likely due to the fast ion release in these glasses (Figure 5) inducing the rise of ionic strength and ionic concentration. For example, B ions have been reported to facilitate antibacterial action, particularly toward *E.coli*. Our results confirmed the composition-dependent antibacterial activity of binary borate BGs [55]. However, further studies are required to elucidate the molecular biology factors that dictate the antibacterial action induced by sol–gel-derived binary borate BGs.

### *In vitro* cytotoxicity and VEGF secretion

**Figure 8.**
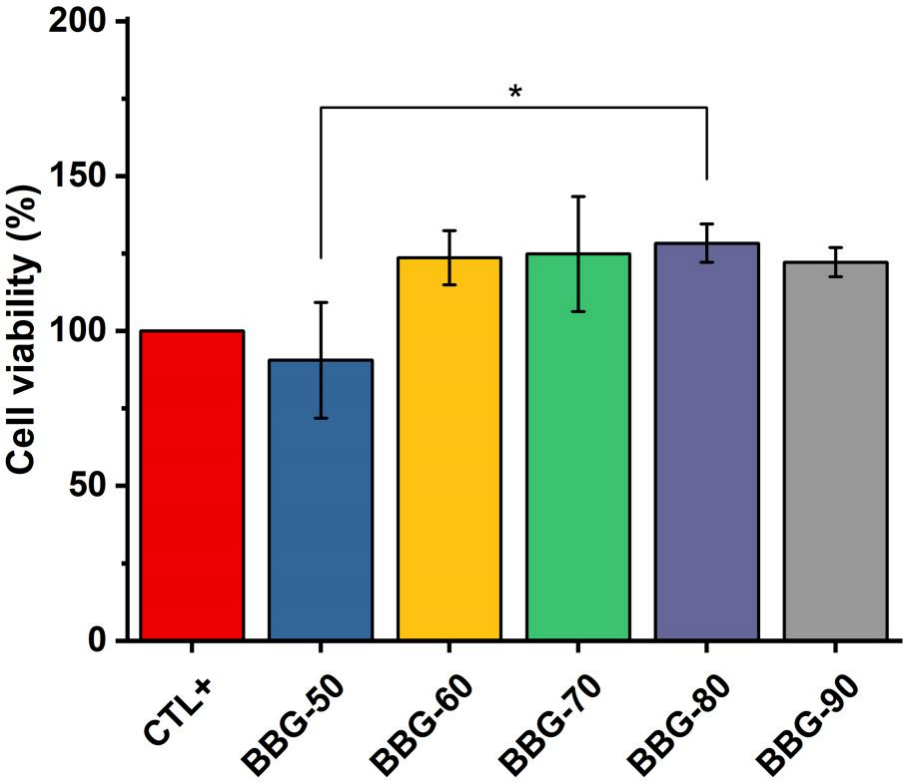
Cell viability of NIH3T3 cells after 24 h of incubation with 1% (w/v) dissolution products of various borate BGs.

Figure 8 shows the cell viability of NIH3T3 cells after culture with the dissolution products of borate BGs. The results showed that BBG-60, BBG-70, BBG-80 and BBG-90 exhibited significantly higher cell viability than that of BBG-50 containing higher calcium content. In addition, the dissolution products of these borate BGs seemed to promote cell viability compared to the control. It is known that Ca ions serve as a signal transduction element that controls multiple cellular functions and play a crucial role in regulating cell proliferation. The fast release of Ca and B ions from borate BGs should contribute to the enhanced cell viability induced by borate BGs. Cell viability and proliferation depend on the concentration of released ions from BGs. This fact could explain the relatively low cell viability induced by BBG-50. Nevertheless, all borate BGs exhibited non-cytotoxicity, confirming their suitability for biomedical applications. Notably, borate BGs exhibited antibacterial activity while being non-cytotoxicity against fibroblasts at the same concentration. This selective toxicity of dissolution products of borate BGs against fibroblasts and bacteria could be explained by the different membrane structures of both cell types and their different sensitivity to dissolution products (mainly boron ions) [56, 57]. However, comprehensive studies are still required to understand the exact mechanism behind the selective toxicity.

The use of boron-containing BGs has aroused some concerns in biomedical applications, due to a generalized view of boron compounds as toxic chemical entities [58]. However, there are also numerous studies reporting the low toxicity risk of using boron-containing compounds, including boron-containing BGs [59]. It is important to understand local toxicity effects of boron-containing BGs and their potential influence on the main organs when these BGs are translated to clinical applications. Recently, a study performed by Zhang *et al*. [59] reported that the implantation of borosilicate BG reinforced PMMA bone cement did not elicit acute systemic toxicity or skin irritation and it did not cause visible damage or weight change to the heart, liver, lung and kidney of rats. The biosafety of the composite bone cement was also verified in large animal models, including rabbits and goats and the results of a first human clinical trial were also favorable. These results evidenced the biosafety of boron-containing BGs. However, the local toxicity of the type of sol–gel-derived borate BGs produced here and their potential toxicity to internal organs have not been investigated, which should be the focus of future *in vivo* studies. In addition, understanding the dose-dependent cytotoxicity of borate BGs is also important, particularly for the design of composites based on combination of borate BGs with other materials. Therefore, the dose-dependent biological properties of borate BGs should be investigated in future studies.

**Figure 9.**
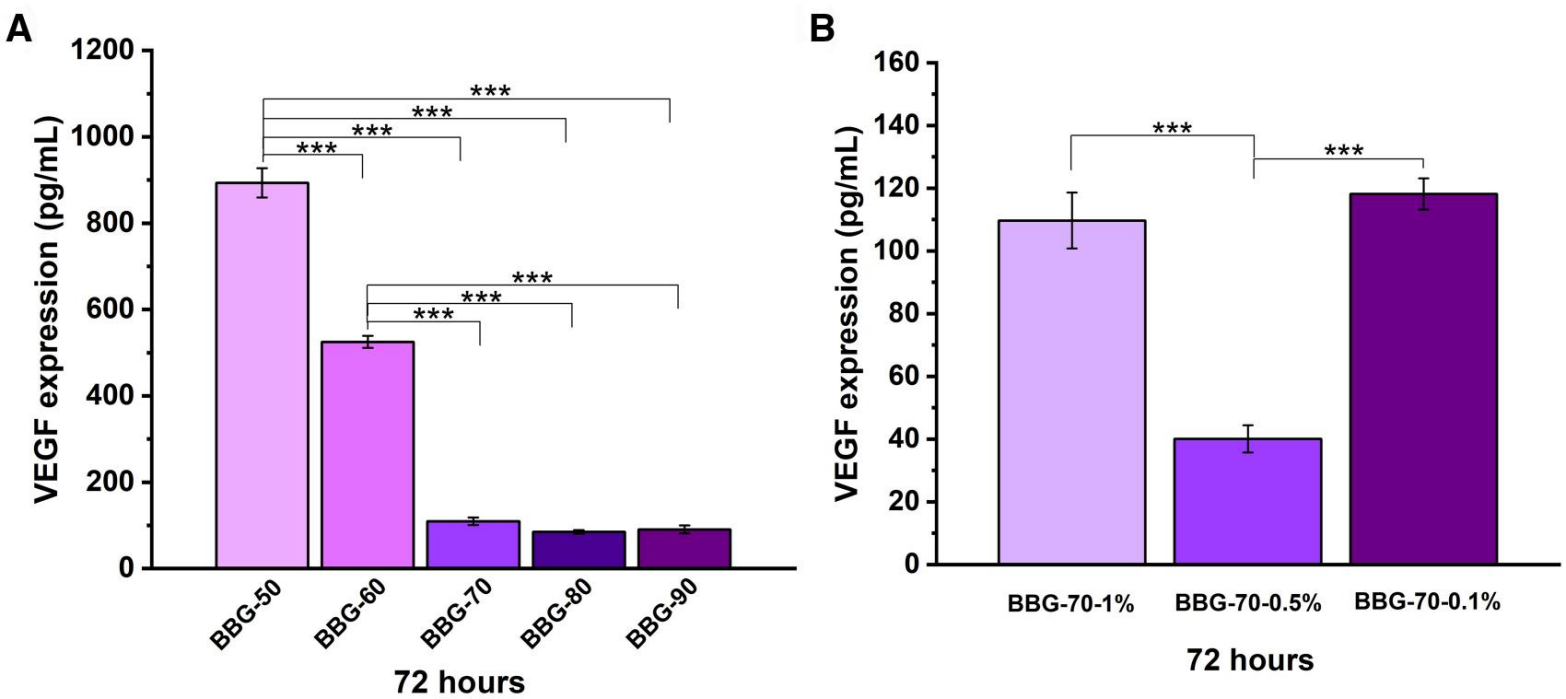
VEGF expression results of binary borate glasses after 72 h of incubation and measured at a wavelength of 450 nm. (**A**) Binary borate BGs; (**B**) BBG-70 1% (w/v), 0.5% (w/v) and 0.1% (w/v) samples.

We also evaluated the expression of VEGF from fibroblast cells stimulated by BGs in order to investigate the effect of borate BG composition on angiogenesis. Figure 9A shows VEGF expression results of cells cultured with dissolution products of borate BGs for 72 h. VEGF concentration (100 pg/ml) remained almost at the same level for BBG-70, BBG-80 and BBG-90. Alternatively, BBG-50 and BBG-60 significantly enhanced VEGF secretion compared to other borate BGs, while BBG-50 (∼900 pg/ml) could stimulate more VEGF secretion than BBG-60 (∼500 pg/ml). We also evaluated the influence of BG concentration on VEGF secretion. As can be seen in Figure 9B, BBG-70 could stimulate VEGF secretion of cells to different extents when used at different concentrations. Concentrations of 1% and 0.1% of BBG-70 could stimulate more VEGF secretion than 0.5% BBG-70. Our results confirmed that a higher concentration of B (Ca) ions seemed to facilitate VEGF secretion as BBG-50 could stimulate almost nine times higher VEGF secretion than BBG-70, BBG-80 and BBG-90. The concentration of borate BGs for specific applications should be carefully considered, as dissolution products could stimulate VEGF secretion to a greater extent at a specific concentration, as observed in the case of BBG-70 used at different concentrations (Figure 9B). Overall, our results indicate that borate BGs can stimulate greater VEGF secretion of mouse fibroblast cells when suitable compositions and doses of BGs are applied.

## Conclusions

In this study, we successfully synthesized binary B_2_O_3_–CaO borate BGs using a sol–gel-based method. Borate BGs with simple composition systems and tunable CaO content could be synthesized. The influence of CaO concentration on morphology, structure and ion release behavior of borate BGs was investigated. The results confirmed that tuning the chemical composition can be used to control the dissolution behavior (ion release) of sol–gel-derived binary borate BGs. We also evaluated the *in vitro* cytotoxicity, hemostatic, antibacterial and angiogenic activities of the newly developed borate BGs. The results indicate that the biological properties of borate BGs are composition-dependent and dose-dependent. Optimizing the composition and concentration allows us to obtain borate BGs that can be used in medical devices, e.g. wound dressings, that can accelerate wound healing by promoting hemostatic, antibacterial and angiogenic performances. Overall, the results of this project provide knowledge regarding the sol–gel synthesis of a new family of borate BGs. The obtained borate BGs can be used to develop novel biomedical devices for chronic wound healing, given their tunable composition, ion release behavior and favorable hemostatic, antibacterial and angiogenic activities.
